# On the Cause and Diagnostic Value of Muscæ Volitantes

**Published:** 1852-09

**Authors:** M. Tavignot


From the Gaz. des Hop., 1851, No. 127.

*On the Cause and Diagnostic Value of Muscæ Volitantes.* By M. TAVIGNOT.

M. TAVIGNOT assigns as the cause of this phenomenon, the passage of the luminous rays through a very circumscribed spot of the semi-transparent tissue of the iris, which has become deprived of its pigmentary matter—a fissure in the uvea. This theory explains: 1st. Why the muscæ are placed near the visual axis, but always on one side of it; 2d. It explains the fact of their disappearance in obscure light, and their especial distinctness in a bright one, which induces the contraction of the pupil, and the enlargement of the aperture in the uvea; 3d. Also their varied form, according to the different action of light upon the eye, and the effect of this upon the size of the fissure; 4th. It explains their appearance after sudden movement of the eyes upwards, which is always accompanied by a contractile oscillation of the iris, as also their diminution or disappearance as the pupil enlarges.

If this theory be sound, the muscæ ought to disappear when the pupil is dilated by belladonna; and M. Tavignot declares that his experiments have convinced him that they do disappear in proportion as artificial mydriasis is thus produced, and that they return again with the returning motions of the iris. It is to be borne in mind that these remarks are referrible only to *essential* muscæ volitantes; M. Tavignot intending to show hereafter, that in the *sympathetic* form (as in glaucoma) an altered condition of the texture of the iris explains the appearance, and adds confirmation to the above view.

*Artificial dilatation of the pupil* enables us to decide whether we have to do with muscæ volitantes, properly so called, or with the spots known as *scotomata,* which are found in partial opacities of the cornea, and in incipient cataract; for while the muscæ volitantes disappear on the production of the mydriasis, the scotomata persist, and even become more distinct.

